# Comparative analysis of oropharyngeal microbiota in healthcare workers post-COVID-19

**DOI:** 10.3389/fcimb.2024.1347345

**Published:** 2024-05-17

**Authors:** Yue Wei, Wenyi Yu, Zhixia Zhang, Siqin Liu, Jianbo Xue, Chunyan Wu, Zhancheng Gao, Shuming Guo

**Affiliations:** ^1^ Nursing of school, Shanxi Medical University, Taiyuan, Shanxi, China; ^2^ Department of Respiratory and Critical Care Medicine, Peking University People’s Hospital, Beijing, China; ^3^ Nursing Department, Linfen Central Hospital, Shanxi, China; ^4^ Department of Prosthodontics, Affiliated Hospital of Stomatology, Nanjing Medical University, Nanjing, Jiangsu, China; ^5^ Institute of Chest and Lung Diseases, Shanxi Medical University, Linfen, Shanxi, China

**Keywords:** COVID-19, oral microbiome, front-line healthcare workers, viral exposure, microbial communities, commensal microorganisms, pathogenic species, oral health

## Abstract

**Background:**

To date, more than 770 million individuals have become coronavirus disease 2019 (COVID-19) convalescents worldwide. Emerging evidence highlights the influence of COVID-19 on the oral microbiome during both acute and convalescent disease phases. Front-line healthcare workers are at an elevated risk of exposure to viral infections, and the effects of COVID-19 on their oral microbiome remain relatively unexplored.

**Methods:**

Oropharyngeal swab specimens, collected one month after a negative COVID-19 test from a cohort comprising 55 healthcare workers, underwent 16S rRNA sequencing. We conducted a comparative analysis between this post-COVID-19 cohort and the pre-infection dataset from the same participants. Community composition analysis, indicator species analysis, alpha diversity assessment, beta diversity exploration, and functional prediction were evaluated.

**Results:**

The Shannon and Simpson indexes of the oral microbial community declined significantly in the post-COVID-19 group when compared with the pre-infection cohort. Moreover, there was clear intergroup clustering between the two groups. In the post-COVID-19 group, the phylum Firmicutes showed a significant increase. Further, there were clear differences in relative abundance of several bacterial genera in contrast with the pre-infection group, including *Streptococcu*s, *Gemella*, *Granulicatella*, *Capnocytophaga*, *Leptotrichia*, *Fusobacterium*, and *Prevotella*. We identified *Gemella* enrichment in the post-COVID-19 group, potentially serving as a recovery period performance indicator. Functional prediction revealed lipopolysaccharide biosynthesis downregulation in the post-COVID-19 group, an outcome with host inflammatory response modulation and innate defence mechanism implications.

**Conclusion:**

During the recovery phase of COVID-19, the oral microbiome diversity of front-line healthcare workers failed to fully return to its pre-infection state. Despite the negative COVID-19 test result one month later, notable disparities persisted in the composition and functional attributes of the oral microbiota.

## 1 Introduction

The global spread of coronavirus disease 2019 (COVID-19), an infectious illness caused by severe acute respiratory syndrome coronavirus 2 (SARS-CoV-2), represents a significant and urgent threat to public health (World Health Organization; Zhu et al., 2020). While most individuals who contract the virus will experience mild to moderate respiratory illness and recuperate without requiring specialized medical care, certain individuals, particularly older adults and those with pre-existing health conditions, may experience severe illness necessitating medical intervention. The virus primarily enters the human body through the angiotensin-converting enzyme 2 (ACE2) and transmembrane serine proteases 2 (TMPRSS2) receptors (Gheblawi et al., 2020; Hoffmann et al., 2020). Because salivary glands and oral mucosal cells express higher levels of ACE2 and TMPRSS2 receptors (Xu et al., 2020a, b; Williams et al., 2021), SARS-CoV-2 enters the human body through the oropharynx, and establishes infection by altering the microbiome and escaping host immunity (Bao et al., 2020). Moreover, the long-lasting impact of COVID-19-induced pathophysiological changes can extend to the microbiome, leading to enduring symptoms (Xiang et al., 2020).

The maintenance of oral microbiota homeostasis is an important component of oral and systemic health. The oral microbiome, including bacteria, fungi, and viruses, is the second largest human microbiome and has been extensively researched (Dewhirst et al., 2010; Baker et al., 2024). Currently, there are nearly 800 bacterial species in the expanded Human Oral Microbiome Database. The development and composition of the oral microbiome depends on a variety of factors, including diseases, personal habits, and environmental factors. Under normal circumstances, the oral microbiome is in a balanced state, which can resist the invasion of external adverse factors on the body and maintain oral health. However, when key factors in the oral environment change, resulting in the competitive growth of opportunistic pathogens such as *Streptococcus*, *Neisseria*, *Corynebacterium*, *Veillonella*, *Gemella*, *Haemophilus*, *Rothia*, and *Porphyromonas* (Li et al., 2014), oral homeostasis is disrupted, which can induce oral and even systemic diseases (Kleinstein et al., 2020). Recently, several studies have shown that SARS-CoV-2 affects the microbiota of the gastrointestinal and upper respiratory tracts (Ma et al., 2021; Ren et al., 2021; Ventero et al., 2021; Wu et al., 2021; Gupta et al., 2022; Ralli et al., 2023). Moreover, the human microbiome is crucial in recovering from various illnesses (Zhang et al., 2015; Chen et al., 2018; Deo and Deshmukh, 2019). During large SARS-CoV-2 outbreaks, many individuals with COVID-19 have demonstrated intricate co-infections involving various pathogens, including some originating from the oral cavity. The connection between the oral microbiome and patients with COVID-19 has been widely described (Bao et al., 2020; Gao et al., 2021; Miller et al., 2021; Ren et al., 2021; Wu et al., 2021). However, the oral microbiome of convalescents remains not thoroughly understood.

Human oral microbes are adaptable and closely related to their surrounding environment. The distinctive chemical and physical parameters delineate a stark contrast between the microbial assemblage within the hospital environment and the surrounding natural milieu (Shobo et al., 2020). Front-line pain-alleviating and life-saving healthcare workers during the COVID-19 pandemic are the most important force in addressing this health crisis. During the fight against COVID-19, healthcare workers exhibited a notably elevated incidence of COVID-19 when contrasted with the general populace. Their extensive engagement with patients, coupled with prolonged exposure to hospital environments, poses a high-risk scenario regarding their microbiome, which remains poorly understood. In view of the characteristics of hospital settings, the study of healthcare workers’ oral microbiome assumes profound significance, both in the context of safeguarding their personal well-being and in the overarching endeavor to control nosocomial infections. Our previous study described the oral microbiome profile of healthcare workers in different clinical scenarios and demonstrated that community diversity, structure, and potential functions differed markedly among departments. Especially in the coronary care unit (CCU), healthcare workers were more likely to exhibit inherent microbiological characteristics (Zhang et al., 2022).

In this study, we analyzed the oral microbiome of healthcare workers post-COVID-19 using 16SrRNA sequencing technology and compared it with the oral microbiome data of healthcare workers pre-infection to elucidate the diversity, composition, and functional characteristics of the oral microbial community in healthcare workers after infection with SARS-CoV-2. This inquiry has substantial implications concerning the well-being of healthcare personnel and the management of nosocomial infections.

## 2 Methods

### 2.1 Study design

This study recruited 55 healthcare workers from Linfen Central Hospital (Shanxi Province, China) from the department of respiratory medicine (RES, n = 18), CCU (n = 10), intensive care unit (ICU, n = 11), and operating room (OR, n = 16). We enrolled individuals who had: (1) participated in the previous study (Zhang et al., 2022); (2) experienced COVID-19 infection, specifically a positive detection of SARS-CoV-2 RNA by reverse transcription-polymerase chain reaction; (3) received a negative COVID-19 test within one month prior to oropharyngeal swab sampling; and (4) worked in the hospital for more than one year. Participants were excluded if they were pregnant or lactating, had any respiratory disease, used antibiotics within 3 months before sampling, or used probiotics within one month prior to sampling. During sampling, the oral hygiene status of the participants was observed according to the World Health Organization oral health standards, which included being clean, without cavities, pain, normal gums, and no bleeding. Those who did not meet these standards were excluded. Probing depth (PD) and bleeding on probing (BOP) was used to evaluate the health status of periodontal tissue. The study was reviewed and approved by the ethics committee of Linfen Central Hospital (Ethics Approval No. 2022-5-1) and all methods were carried out in accordance with relevant guidelines and regulations. All participants signed informed consent prior to participating.

### 2.2 Sample collection

Demographic data were collected using questionnaires. Oropharyngeal swab sampling occurred 1 month after a negative COVID-19 test. To ensure that the samples were not contaminated by food or drink, the participants were required to refrain from eating or drinking for 3h prior to sampling. To guarantee sample quality, the operator used a sampling swab to gently rub both sides of the participant’s pharyngeal tonsils in a back-and-forth motion at least 3 times, and then wiped the posterior pharyngeal wall in an up-and-down motion at least 3 times. Further, all fresh oropharyngeal swabs were collected by one nurse who received professional training and they were stored at -80 °C immediately prior to DNA extraction.

### 2.3 DNA extraction and PCR amplification

The extraction of total genomic DNA from oropharyngeal swabs was performed using the TIANamp Swab DNA Kit (TIANGEN, China). The Ion 16S™ Metagenomics Kit (ThermoFisher Scientific, USA) was used for amplifying the hypervariable regions (V2, V3, V4, V6–7, V8, and V9) of the 16S rRNA. To avoid possible interference, two distinct primer pools were prepared for each DNA template following the manufacturer’s instructions. Subsequently, we merged the PCR products of two separate tubes for further amplicon purification using XP reagent and they were quantified using Qubit4 (ThermoFisher Scientific, USA).

### 2.4 Library preparation and sequencing

Following amplification, PCR products were purified and end-repaired for barcode ligation. Library amplification and pooling for template preparation was done in equimolar amounts using Ion Chef according to the Ion 530 Kit-Chef protocol. The libraries were pooled to obtain a final concentration of 25 pM. Sequencing of the amplicon libraries was carried out on a 530 Chip using the Ion Torrent S5 system (Thermo Fisher, USA). All amplified regions were sequenced, and multiple variable regions were included in the sequencing approach to achieve enhanced resolution.

### 2.5 Bioinformatic analysis

Quality filtering, trimming, and dereplication of the raw reads was completed using the Ion Reporter metagenomics workflow with default parameters. Next, the UCHIME algorithm (Edgar et al., 2011)was used to remove the chimeric sequences and the UNOISE3 algorithm (Edgar, 2016) was used to generate denoising amplicon sequence variants. Based on vsearch (Rognes et al., 2016), we completed taxonomic assignment referring to the SILVA (V 138.1) (Glockner et al., 2017) and GreenGene (DeSantis et al., 2006) databases with a threshold of 97%. “Unassigned” refers to readings that cannot find matching sequences in the reference database during the alignment process. “Other” refers to a grouping of bacterial genera that, while capable of being matched to a reference database, do not fall within the top 20 most abundant categories. Alpha diversity was evaluated using the Shannon, Simpson, Chao 1, richness, and abundance-based coverage estimator (ACE) indexes. Beta diversity was evaluated by permutational multivariate analysis of variance (PERMANOVA) based on the Bray−Curtis distance. Next, linear discriminant analysis (LDA) effect size (LEfSe) (Segata et al., 2011) was employed, applying the criteria of LDA > 2 and P < 0.05, to identify differential bacterial taxa across groups. To assess the differences in the microbiome, we conducted statistical analysis and qualitative and quantitative analyses of the microbiome data using the web-based tool MicrobiomeAnalyst (Lu et al., 2023).

Additionally, PICRUSt2 (Douglas et al., 2020) was utilized to identify microbiome-associated pathways based on the inferred metagenomes of taxa. It predicts functional abundance based on marker gene sequences. Metabolic pathway data were compared using two-sided Welch’s t-test, followed by rigorous filtration for false discoveries through the Benjamini–Hochberg method. To aid in determining features with biologically relevant differences between groups, we set a confidence interval of 95% and effect size > 0.05. Items with q-values < 0.05 were considered significant. The statistical analysis and visualization of functional data were performed using the STAMP software (Parks et al., 2014).

### 2.6 Statistical analysis

Statistical analyses were performed using SPSS v20.0 for Windows. Continuous variables between two groups were compared by Student’s t-test or Wilcoxon rank-sum test. Normally distributed values are presented as mean ± standard deviation, and nonnormally distributed values are presented as the median (interquartile range). Categorical variables between two groups were compared by the *X* ^2^ test or Fisher’s exact test. Differences among three groups were evaluated by one-way analysis of variance. A significance threshold of *P* < 0.05 (two-tailed) was established.

## 3 Results

### 3.1 Characterization of study participants

The participants provided oropharyngeal swabs both before and after contracting COVID-19. The demographic characteristics of the study cohort are detailed in **Table 1**. The cohort exhibited an average age of 32.76 years, with 78.18% being female and 60% comprising nurses. The mean cumulative years of professional experience was 8.82. Notably, the study participants had no pre-existing periodontal diseases and other comorbidities that might influence the oral microbiome. Furthermore, all participants had received COVID-19 vaccination.

**Table 1:** Demographic data of study participants.

Chracteristics	Total (n=65)	RES (n=18)	CCU (n=10)	ICU (n=11)	OR (n=16)	*P* value
Age	32.76 ± 4.81	33.83 ± 4.88	29.70 ± 2.79	33.00 ± 4.82	33.31 ± 5.33	0.1355^a^
Gender						0.1109^b^
Male	12 (21.8%)	2 (11.1%)	1 (10.0%)	2 (18.2%)	7 (43.8%)	
Female	43 (78.2%)	16 (88.9%)	9 (90%)	9 (81.8%)	9 (56.2%)	
Position						0.1355^b^
Doctor	22 (40%)	9 (50%)	1 (10%)	6 (54.5%)	6 (37.5%)	
Nurse	33 (60%)	9 (50%)	9 (90%)	5 (45.5%)	10 (62.5%)	
Seniority	8.82 ± 4.83	8.94 ± 5.76	6.00 ± 2.67	10.4 ± 4.01	9.31 ± 4.90	0.1469^a^
Comorbidity	NA	–	–	–	–	1^b^
BOP (-)	55 (100%)	18 (100%)	10 (100%)	11 (100%)	16 (100%)	1^b^
PD	2.26 ± 0.43	2.27 ± 0.44	2.39 ± 0.45	2.20 ± 0.46	2.23 ± 0.43	0.7462^a^
Vaccination	55 (100%)	18 (100%)	10 (100%)	11 (100%)	16 (100%)	1^b^

P value were calculated using a, kruskal-wallis test; b, chi-square test.

### 3.2 Distinct oral microbial structure and diversity between pre-infection and post-COVID-19 groups

**Figure 1:**
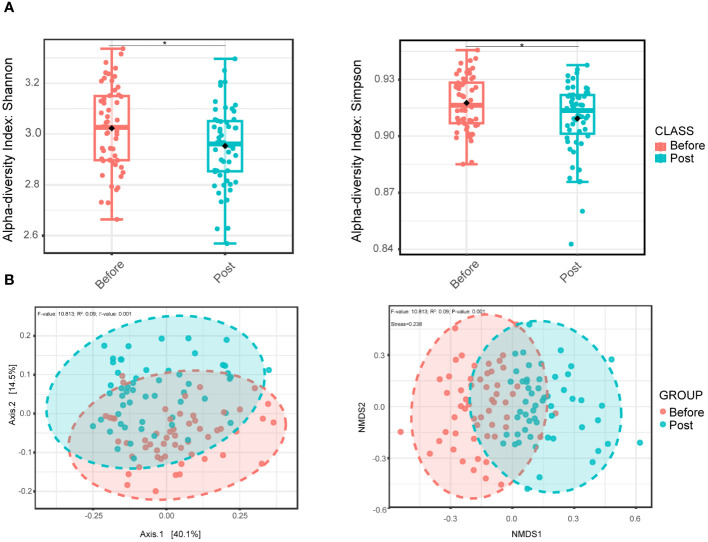
Oral bacterial microbial diversity of before and post COVID-19 infection group. **(A)** Alpha diversity boxplot of the oral microbial community based on the Shannon index (left, wilcoxon test, *P* = 0.041) and Simpson index (right, wilcoxon test, *P* = 0.038), representing differences in within-sample diversity between two groups. **(B)** PCoA plot (left) based on Bray-Curtis dissimilarity and with PERMANOVA statistics depicting significant differences (*P*-value < 0.001). NMDS plot (right) based on Bray-Curtis dissimilarity and PERMANOVA statistics showing significant difference (*P*-value < 0.001) between the two groups. * *P <*0.05.

Initially, we evaluated the alpha diversity between the two groups. Compared to the pre-infection group, the post-COVID-19 group exhibited a lower level of oral microbial diversity in the Shannon and Simpson indexes, and the differences were statistically significant (**Figure 1A**). Nevertheless, no noteworthy distinctions were observed between the two groups concerning the richness, Chao 1, and ACE indexes (**Supplementary Figure S1**). In addition, beta diversity indicated distinct intergroup clustering of oral microbial communities by principal coordinate and nonmetric multidimensional scaling analyses (**Figure 1B**).

### 3.3 Characterization of oral microbiome in the post-COVID-19 group

**Figure 2:**
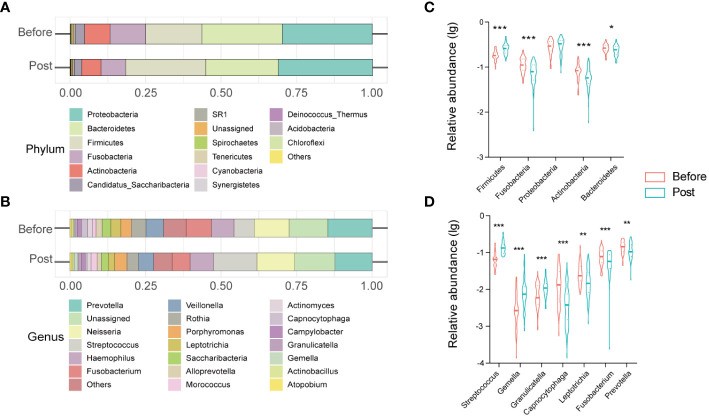
Structure and alteration of the oral microbiome in healthcare workers from before and post COVID-19 groups. Stacked bar charts show the relative abundance of before and post groups at the phylum level **(A)** and at the genus level **(C)**. Violin charts reveal significant differences in the relative abundance of oral microbiota at the phylum level **(B)** and at the genus level **(D)**. * *P* < 0.05; ** *P* < 0.01; *** *P*< 0.001.

The oral microbiome profile of the post-COVID-19 group comprised 13 phyla, 20 classes, 35 orders, 64 families, and 121 genera. Core phyla were defined as those identified in all samples. Eleven core phyla were identified in the post-COVID-19 samples, including Proteobacteria, Firmicutes, Bacteroidetes, Fusobacteria, Actinobacteria, Candidatus_Saccharibacteria, SR1, Spirochaetes, Tenericutes, Cyanobacteria, and Synergistetes (**Figure 2A**). The top 10 genera included *Prevotella*, *Neisseria*, *Streptococcus*, *Haemophilus*, *Fusobacterium*, *Veillonella*, *Rothia*, *Porphyromonas*, *Leptotrichia*, and *Saccharibacteria* (**Figure 2B**).

Furthermore, we clarified the bacterial composition and variations in the oral microbiome between the pre-infection and the post-COVID-19 groups. At the phylum level, Proteobacteria, Bacteroidetes, Firmicutes, Fusobacteria, and Actinobacteria accounted for > 95% of sequences on average and comprised the five most abundant bacteria in the pre-infection and post-COVID-19 groups. Further, in the post-COVID-19 group, there was a statistically significant increase in the relative abundance of Firmicutes (*P* < 0.0001), accompanied by decreased relative abundances of Bacteroidetes, Fusobacteria, and Actinobacteria compared to the pre-infection group (**Figure 2C**). At the genus level, the genera *Prevotella*, unassigned, *Neisseria*, *Streptococcus*, *Haemphilus*, *Fusobacterium*, Others, *Veillonella*, *Rothia*, and *Porphyromonas*, were the ten most dominant bacteria, accounting for > 80% of the total in both groups. Among the top 20 genera, analysis of differential expression using the Mann–Whitney U test uncovered seven genera that exhibited variations between the two groups. Three genera (*Streptococcus*, *Gemella*, and *Granulicatella*) exhibited significant increases (*P* < 0.001), whereas four genera (*Capnocytophaga*, *Leptotrichia*, *Fusobacterium*, and *Prevotella*) displayed significant reductions in the post-COVID-19 group (**Figure 2D**).

**Figure 3:**
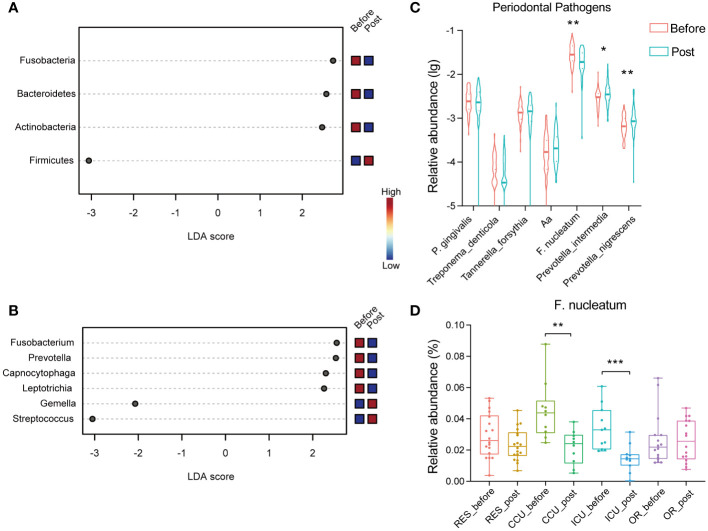
Dot plots show the significant differences in relative abundance (LDA > 2) between two groups via LEfSe analysis at the phylum level **(A)** and genus level **(B)**. **(C)** The violin chart reveals the differences in the relative abundance of periodontal pathogens in healthcare workers between two groups. **(D)** The box plot shows the relative abundance of F. nucleatum (CCU_before vs. CCU_post *P* =0.003; ICU_before vs. ICU_post *P*=0.0006) across department between before and post group. (Aa, *Aggregatibacter actinomycetemcomitans*). * *P* < 0.05; ** *P* < 0.01; *** *P* < 0.001.

Further, we carried out LEfSe analysis to pinpoint the specific main microbiota that differed significantly between the groups, using a cut-off value with an LDA score > 2. As illustrated in **Figure 3A**, the phylum Firmicutes was enriched in the post-COVID-19 group, while Actinobacteria, Bacteroidetes, and Fusobacteria were enriched in the pre-infection group. At the genus level, the relative abundances of *Streptococcus* and *Gemella*, both belonging to the phylum Firmicutes, were clearly higher in the post-COVID-19 group as compared with the pre-infection group (**Figure 3B**). Conversely, *Leptotrichia*, *Capnocytophaga*, *Prevotella*, and *Fusobacterium* showed an inverse trend. There were also significant differences at the species level of *Gemella* post-COVID-19, including *Gemella sanguinis*, *Gemella haemolysans*, and *Gemella morbillorum* (**Supplementary Figure S2**).

### 3.4 Differences in periodontal disease related microorganisms between the two groups

The oral microbiota composition plays a significant role in influencing oral health and oral diseases. We further investigated the microbial changes associated with periodontal disease within the post-COVID-19 group. Among more than 700 bacterial species living in the oral cavity, a bacterial complex named the “red complex” made up of *Porphyromonas gingivalis*, *Treponema denticola*, and *Tannerella forsythia* has been strongly associated with periodontal lesions. In our study, when comparing the post-COVID-19 group to the pre-infection group, there were no statistically significant differences observed in the “red complex”. Similarly, the relative abundance variation of *Aggregatibacter actinomycetemcomitans* that is associated with periodontal disease was not significant. However, the relative abundance of some microbiota related to oral diseases had changed. The relative abundances of *Prevotella intermedia* and *Prevotella nigrescens*, associated with inflammatory periodontitis, also displayed significant increases (**Figure 3C**). Interestingly, in the post-COVID-19 group, there was a significant decrease in the relative abundance of *Fusobacterium nucleatum*, particularly in the CCU and ICU groups (**Figure 3D**).

### 3.5 Correlation between oral microbiota and manifestations of COVID-19

**Figure 4:**
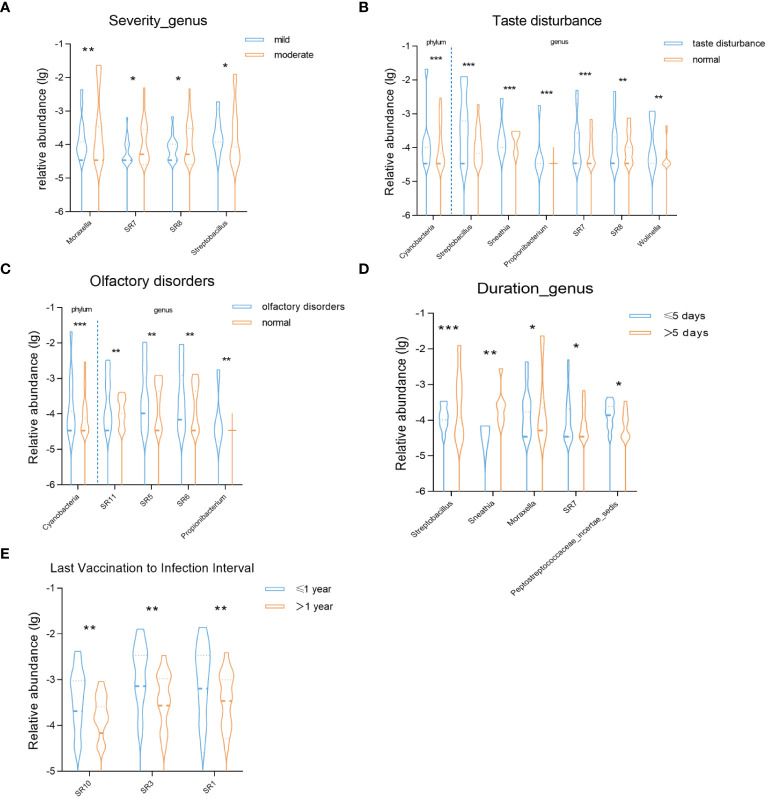
Violin charts reveal significant differences in the relative abundance of oral microbiota **(A)** between mild and moderate groups at the genus level, **(B)** between taste disturbance and normal groups at the phylum and genus levels, **(C)** between olfactory disorders and normal groups at the phylum and genus levels, **(D)**between duration of infection ≤5 days and >5 days groups at the genus level, **(E)** between the interval from the last dose of vaccine before infection to infection ≤1 year and >1 year groups at the genus level. * *P* < 0.05; ** *P* < 0.01; *** *P*< 0.001.

First, we compared the correlation between the severity of COVID-19 infection and the oral microbiota during the recovery period. In the moderate symptom group, we observed the enrichment of four bacterial genera: Moraxella, SR7, SR8, and Streptobacillus (**Figure 4A**). Subsequently, we conducted a comparative analysis based on symptom grouping, specifically focusing on taste disturbance and olfactory disorders. Notably, both groups exhibited enrichment of the phylum Cyanobacteria (**Figures 4B, C**). At the genus level, Streptobacillus, Sneathia, Prolionibacterium, SR7, SR8, and Wolinella were enriched in the taste disturbance group, while SR11, SR5, SR6, and Prolionibacterium were enriched in the olfactory disorders group (**Figures 4B, C**). Furthermore, we analyzed the impact of the duration of COVID-19 infection on the oral microbiota during the recovery period. Based on our data, we categorized the patients into two groups: those with an infection duration of less than or equal to 5 days and those with a duration of more than 5 days. In the group with a duration of more than 5 days, Streptobacillus, Sneathia, and Moraxella were enriched, while SR7 and Peptostreptococcaceae incertae sedis were enriched in the group with a duration of less than or equal to 5 days. Additionally, we analyzed the impact of the time interval between the last shot of vaccine and COVID-19 infection on oral microbiota during the recovery period (**Figure 4D**). Our findings revealed that in the group with a time interval of less than or equal to one year, the bacterial genera SR10, SR3, and SR1 were enriched (**Figure 4E**).

### 3.6 Function prediction

**Figure 5:**
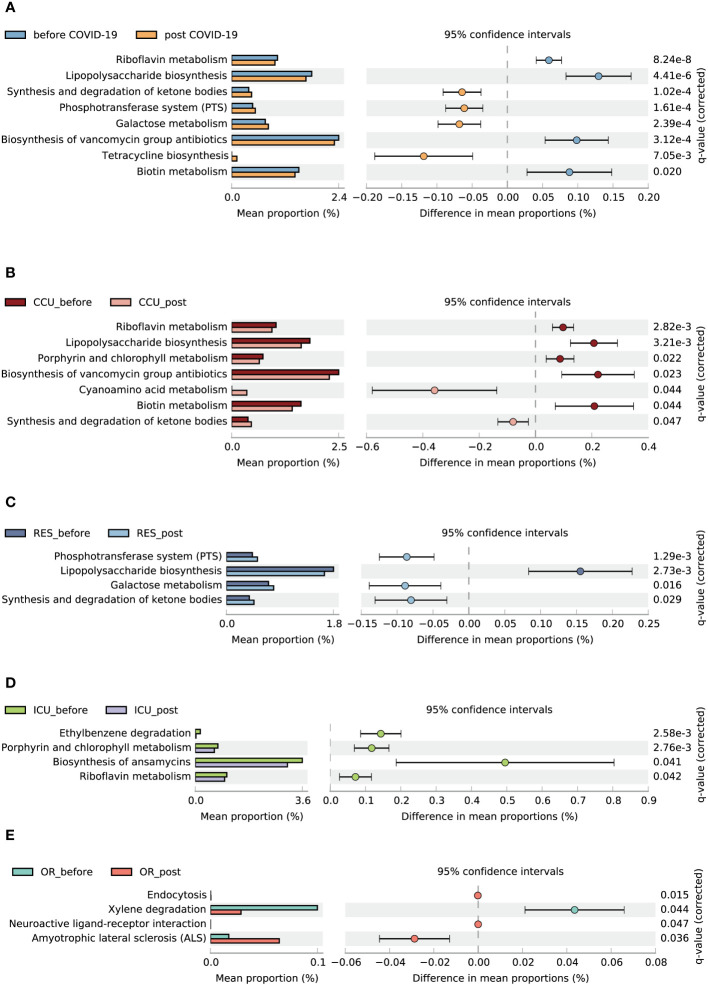
Functional characterization of different groups based on PICRUSt2 analysis. Bar charts show the functional differences between the before and post COVID-19 group **(A)**, CCU_bofore and CCU_post **(B)**, ICU_before and ICU_post **(C)**, OR_before and OR_post **(D)**, RES_before and RES_post **(E)**. Data were compared by two-sided Welch’s t–test and filtered for false discoveries using the Benjamini-Hochberg method. Items with q-values < 0.05 and effect size > 0.05 are shown in the figure.

A total of 162 metabolic pathways were annotated. Eight distinct pathways showed disparity between the pre-infection and post-COVID-19 groups. Compared to the pre-infection group, four related to synthesis and degradation of ketone bodies, the phosphotransferase system (PTS), galactose metabolism, and tetracycline biosynthesis were enriched in the post-COVID-19 group. Nevertheless, riboflavin metabolism, lipopolysaccharide (LPS) biosynthesis, biosynthesis of vancomycin group antibiotics, and biotin metabolism were downregulated in the post-COVID-19 group (**Figure 5A**). Through subgroup analysis, we also found that the CCU and RES made major contributions to metabolic changes between the pre-infection and post-COVID-19 groups (**Figures 5B–E**).

## 4 Discussion

COVID-19 has caused a profound global impact. Even after a negative COVID-19 test, most patients with COVID-19 still experience a long recovery period. We compared the oral microbiome of oropharyngeal swab samples collected from healthcare workers pre-infection and post-COVID-19. A particularly significant discovery was that the oral microbiome did not fully revert to its normal state during recovery. To our knowledge, this study was the first exploration of the oral microbiome in healthcare workers in the hospital environment during the recovery period of COVID-19.

Our results suggested that there were significant differences in oral microbial diversity between the pre-infection and post-COVID-19 groups. Several studies (Gao et al., 2021; Ma et al., 2021) have shown that the oral microbial diversity in individuals with confirmed COVID-19 is lower than that of healthy controls. Therefore, our present study implied that the oral microbial diversity of our participants had not fully returned to the healthy level. The study revealed a clear reduction in oropharyngeal microbial diversity in the post-COVID-19 group, consistent with prior research (Gao et al., 2021; Cui et al., 2022; Wei et al., 2023), thus emphasizing the existence of oropharyngeal microbial dysbiosis.

Additionally, we observed substantial alterations in both the composition and abundance of the oral microbiome after SARS-CoV-2 infection. In contrast to the pre-infection cohort, the post-COVID-19 group exhibited a decline in the relative abundance of genera such as *Fusobacterium*, *Prevotella*, *Capnocytophaga*, and *Leptotrichia*. In contrast, *Gemella* and *Streptococcus*, genera within the phylum Firmicutes, displayed an increased trend in the post-COVID-19 population. Several studies (Gao et al., 2021; Wei et al., 2023) have demonstrated an elevation in the butyrate-producing bacteria *Fusobacterium*, and a reduction in the opportunistic pathogens *Leptotrichia* and *Prevotella* in the recovery of patients with COVID-19. During the recovery process, the augmentation of butyrate-producing microorganisms has the potential to facilitate the restoration of the intestinal mucosal barrier and contribute to the healthy status of distant organs such as the lungs, thereby assuming a pivotal role in anti-inflammatory mechanisms (Louis and Flint, 2009; Gautier et al., 2021). It has been reported that butyrate can be used as a probiotic for the prevention and treatment of COVID-19 (Gautier et al., 2021; K et al., 2021; Paparo et al., 2022). Regarding *Fusobacterium*, our findings indicated a decrease in its abundance and did not align with those observed in previous studies; however, in terms of oral opportunistic pathogens, the outcome was uncontroversial. The reduction in *Fusobacterium* prevalence was the recovery period performance indicator. Concerning our differences from previous research, we concluded that the participants were situated at disparate stages of the recovery trajectory. Consequently, this speculation requires confirmation through analysis of further samples from patients across various recovery stages. High levels of LPS, potentially produced by *Leptotrichia*, can initiate the systemic proinflammatory phase by activating toll-like receptors (TLR) (Ren et al., 2021; Yeoh et al., 2021). This activation results in the release of cytokines and reactive oxygen species, thereby mediating acute lung injury. TLRs play an important role in the occurrence and development of COVID-19 and may be potential targets for disease treatment and COVID-19 vaccine (Aboudounya and Heads, 2021; Khanmohammadi and Rezaei, 2021; Liu et al., 2022). Meanwhile, *Prevotella* has the potential to stimulate inflammation, attenuate innate immune reactions, and contribute to oral inflammatory processes (Kononen et al., 2022). Overall, our findings indicated a distinctive oral bacterial microbiota composition in individuals who had recovered from COVID-19, which was characterized by different bacterial abundances.

LEfSe analysis showed that *Streptococcus* and *Gemella* were the predominant genera in the post-COVID-19 group. Despite *Streptococcus* not being classically associated with respiratory infections, certain Streptococcus species have been implicated as causative agents in pulmonary infections (Akata et al., 2016). *Streptococcus suis and S. agalactiae* stimulated the expression of ACE2 of Vero cells *in vitro*, which may promote SARS-CoV-2 infection (Xiong et al., 2021). *Gemella* are Gram-positive, facultatively anaerobic bacteria indigenous to the oral cavity, characterized by haemolytic activity. Levels of *Gemella* spp. or *Gemella haemolysans* are elevated in healthy controls compared to that in individuals with periodontitis (Kirst et al., 2015; Arredondo et al., 2020). Additionally, proteins secreted by *G. haemolysans* exert a direct inhibitory effect on the growth of *P. gingivailis*, which is one of the major pathogens directly responsible for development of chronic periodontitis in humans and dental plaque colonization in the oral cavity, suggesting that *G. haemolysans* is associated with a healthy oral environment. Miyoshi et al. introduced *G. haemolysans* as a promising probiotic candidate for the treatment of periodontal disease (Miyoshi et al., 2021). Further, several studies (Bainbridge and Darveau, 2001) indicated that LPS, as a virulence factor, influences *P. gingivalis* activity. In our study, the variation of LPS was also closely related to periodontal diseases. Our previous study (Zhang et al., 2022) described the relative abundance of *F. nucleatum* as approximately 10× higher than that of *P. gingivalis*. However, the relative abundance of *F. nucleatum* in the CCU and ICU groups reduced significantly post-COVID-19, indicating that the periodontal disease risk also changed post-COVID-19. In short, the changes in these bacteria demonstrated that the oral microbiome of healthcare workers in the recovery period was in a state of disorder.

Our study results revealed potential complex relationships among COVID-19 infection severity, symptoms, duration of infection, vaccination status, and changes in oral microbiota during the recovery period. Previous research has found that the severity of the disease during COVID-19 infection significantly influences the respiratory microbiota (Kim et al., 2023). Our findings emphasize significant enrichment of specific bacterial genera among patients with moderate symptoms. Analysis based on taste and olfactory disturbance symptoms highlighted the enrichment of Cyanobacteria at the phylum level in both groups, underscoring potential links between these bacterial taxa and chemosensory dysfunction. Cyanobacteria are a type of gram-negative bacteria used for photosynthesis and are present in all terrestrial and aquatic ecosystems. There is evidence suggesting that cyanobacteria and their metabolites may indirectly impact human health through interactions with the gut microbiota and immune system (Hu and Rzymski, 2022). Certain cyanobacterial metabolites have been shown to alter the composition of the gut microbiota, potentially affecting host health and susceptibility to disease (Buratti et al., 2017). Differences at the genus level also indicate subtle relationships between sensory symptom manifestation and oral microbiota composition during the recovery period of COVID-19 patients. Additionally, vaccination status can influence oral microbiota (Uehara et al., 2022), as indicated by potential interactions between vaccination status and oral microbiota composition in our study.

Several functional pathways, such as degradation of ketone bodies, the PTS and galactose metabolism, were significantly increased in post-COVID-19 group. Hirschberger S et al. found that ketone bodies improve human CD8+ cytotoxic T-cell immune response during COVID-19 infection, indicating the beneficial role of ketone bodies in the disease process (Hirschberger et al., 2022). PTS not only catalyzes transport but also has various regulatory functions. It plays a role in carbon and nitrogen metabolism, regulates iron and potassium levels affecting pathogen virulence, and mediates stress responses (Kotrba et al., 2001). Meanwhile, these enrichment pathways are all related to energy metabolism (Kotrba et al., 2001; Rui, 2014; Coelho et al., 2015). Evidence suggests that COVID-19 disease can cause an energy supply deficit in patients (Ozilgen and Yilmaz, 2021). Consequently, we prudently suggest that the activation of energy metabolism pathways may potentially contribute to facilitating the recovery process from the disease. The downregulation of certain metabolic pathways may hold potential benefits for the recuperation process following infection. Riboflavin, commonly known as vitamin B2, is closely related to energy metabolism, enhancing immune function, and preventing and alleviating inflammatory reactions (Thakur et al., 2017). Further, LPS, a key inflammatory mediator, has been proposed to function as an important molecule that alerts the host of potential bacterial infection because of its ability to potently activate host inflammatory and innate defence responses (Teixeira et al., 2021). Collectively, these findings tentatively implied that the oral bacterial metabolism of healthcare workers underwent dynamic alterations throughout the recovery phase, including key aspects such as the regulation of vitamin metabolic pathways, modulation of inflammatory responses and the immune functions. However, it is important to note that further investigation is necessary to fully elucidate the underlying mechanisms and the exact nature of these alterations.

In this study, we collected oropharyngeal swabs from the same population both before and post-COVID-19 and participants had no other diseases affecting the oral microbiota at the time of sampling. To mitigate related factors affecting the oral microbiota, we obtained comprehensive participant demographic information. Nonetheless, this study had several limitations. First, despite our longitudinal study design, we still lacked long-term tracking and monitoring considering the dynamic changes in oral microbiota with recovery. Second, we did not describe the characteristics of nonbacterial microorganisms (such as fungi, viruses, and archaea) that also play an important role in the oral cavity. Further, the study was conducted within a single center, raising the possibility of limited generalizability to other populations. It remains uncertain whether similar alterations in oral microbiota are observable among convalescents in other medical institutions and general population.

## 5 Conclusion

In conclusion, our research clarified that the oral microbial diversity of front-line healthcare workers showed meaningful differences during the recovery phase from COVID-19. There were still notable changes in the composition of oral microbiota and function profiles among healthcare workers even one month after the negative COVID-19 test result. These findings underscore the importance of monitoring and maintaining the oral health of healthcare professionals, particularly during pandemic situations.

## Data availability statement

The datasets presented in this study can be found in online repositories. The names of the repository/repositories and accession number(s) can be found below: http://www.ncbi.nlm.nih.gov/bioproject/1038769, with the corresponding BioProject ID of PRJNA1038769.

## Ethics statement

The studies involving humans were approved by the ethics committee of Linfen Central Hospital. The studies were conducted in accordance with the local legislation and institutional requirements. The participants provided their written informed consent to participate in this study.

## Author contributions

YW: Writing – original draft, Writing – review & editing. WY: Data curation, Methodology, Writing – original draft, Writing – review & editing. ZZ: Methodology, Writing – review & editing. SL: Methodology, Writing – review & editing. JX: Data curation, Methodology, Writing – review & editing. CW: Methodology, Writing – review & editing. ZG: Data curation, Methodology, Writing – review & editing. SG: Data curation, Funding acquisition, Methodology, Writing – review & editing.

## References

[B1] Aboudounya M. M. Heads R. J. (2021). COVID-19 and toll-like receptor 4 (TLR4): SARS-coV-2 may bind and activate TLR4 to increase ACE2 expression, facilitating entry and causing hyperinflammation. Mediators Inflammation 2021, 8874339. doi: 10.1155/2021/8874339 PMC7811571 33505220

[B2] Akata K. Yatera K. Yamasaki K. Kawanami T. Naito K. Noguchi S.. (2016). The significance of oral streptococci in patients with pneumonia with risk factors for aspiration: the bacterial floral analysis of 16S ribosomal RNA gene using bronchoalveolar lavage fluid. BMC Pulm Med. 16, 79. doi: 10.1186/s12890-016-0235-z 27169775 PMC4864928

[B3] Arredondo A. Blanc V. Mor C. Nart J. Leon R. (2020). Tetracycline and multidrug resistance in the oral microbiota: differences between healthy subjects and patients with periodontitis in Spain. J. Oral. Microbiol. 13, 1847431. doi: 10.1080/20002297.2020.1847431 33391624 PMC7717685

[B4] Bainbridge B. W. Darveau R. P. (2001). Porphyromonas gingivalis lipopolysaccharide: an unusual pattern recognition receptor ligand for the innate host defense system. Acta Odontol Scand. 59, 131–138. doi: 10.1080/000163501750266710 11501881

[B5] Baker J. L. Mark Welch J. L. Kauffman K. M. McLean J. S. He X. (2024). The oral microbiome: diversity, biogeography and human health. Nat. Rev. Microbiol. 22, 89–104. doi: 10.1038/s41579-023-00963-6 37700024 PMC11084736

[B6] Bao L. Zhang C. Dong J. Zhao L. Li Y. Sun J. (2020). Oral microbiome and SARS-coV-2: beware of lung co-infection. Front. Microbiol. 11. doi: 10.3389/fmicb.2020.01840 PMC7411080 32849438

[B7] Buratti F. M. Manganelli M. Vichi S. Stefanelli M. Scardala S. Testai E.. (2017). Cyanotoxins: producing organisms, occurrence, toxicity, mechanism of action and human health toxicological risk evaluation. Arch. Toxicol. 91, 1049–1130. doi: 10.1007/s00204-016-1913-6 28110405

[B8] Chen C. Hemme C. Beleno J. Shi Z. J. Ning D. Qin Y.. (2018). Oral microbiota of periodontal health and disease and their changes after nonsurgical periodontal therapy. ISME J. 12, 1210–1224. doi: 10.1038/s41396-017-0037-1 29339824 PMC5932080

[B9] Coelho A. I. Berry G. T. Rubio-Gozalbo M. E. (2015). Galactose metabolism and health. Curr. Opin. Clin. Nutr. Metab. Care 18, 422–427. doi: 10.1097/MCO.0000000000000189 26001656

[B10] Cui G. Y. Rao B. C. Zeng Z. H. Wang X. M. Ren T. Wang H. Y.. (2022). Characterization of oral and gut microbiome and plasma metabolomics in COVID-19 patients after 1-year follow-up. Mil Med. Res. 9, 32. doi: 10.1186/s40779-022-00387-y 35715833 PMC9204369

[B11] Deo P. N. Deshmukh R. (2019). Oral microbiome: Unveiling the fundamentals. J. Oral. Maxillofac. Pathol. 23, 122–128. doi: 10.4103/jomfp.JOMFP_304_18 PMC6503789 31110428

[B12] DeSantis T. Z. Hugenholtz P. Larsen N. Rojas M. Brodie E. L. Keller K.. (2006). Greengenes, a chimera-checked 16S rRNA gene database and workbench compatible with ARB. Appl. Environ. Microbiol. 72, 5069–5072. doi: 10.1128/AEM.03006-05 16820507 PMC1489311

[B13] Dewhirst F. E. Chen T. Izard J. Paster B. J. Tanner A. C. Yu W. H.. (2010). The human oral microbiome. J. Bacteriol 192, 5002–5017. doi: 10.1128/JB.00542-10 20656903 PMC2944498

[B14] Douglas G. M. Maffei V. J. Zaneveld J. R. Yurgel S. N. Brown J. R. Taylor C. M.. (2020). PICRUSt2 for prediction of metagenome functions. Nat. Biotechnol. 38, 685–688. doi: 10.1038/s41587-020-0548-6 32483366 PMC7365738

[B15] Edgar R. C. (2016). UNOISE2: improved error-correction for Illumina 16S and ITS amplicon sequencing. bioRxiv 57, 08–12. doi: 10.1101/081257

[B16] Edgar R. C. Haas B. J. Clemente J. C. Quince C. Knight R. (2011). UCHIME improves sensitivity and speed of chimera detection. Bioinformatics 27, 2194–2200. doi: 10.1093/bioinformatics/btr381 21700674 PMC3150044

[B17] Gao M. Wang H. Luo H. Sun Y. Wang L. Ding S.. (2021). Characterization of the human oropharyngeal microbiomes in SARS-coV-2 infection and recovery patients. Adv. Sci. (Weinh) 8, e2102785. doi: 10.1002/advs.202102785 34423593 PMC8529429

[B18] Gautier T. David-Le Gall S. Sweidan A. Tamanai-Shacoori Z. Jolivet-Gougeon A. Loreal O.. (2021). Next-generation probiotics and their metabolites in COVID-19. Microorganisms 9 (5), 941. doi: 10.3390/microorganisms9050941 33925715 PMC8146258

[B19] Gheblawi M. Wang K. Viveiros A. Nguyen Q. Zhong J. C. Turner A. J.. (2020). Angiotensin-converting enzyme 2: SARS-coV-2 receptor and regulator of the renin-angiotensin system: celebrating the 20th anniversary of the discovery of ACE2. Circ. Res. 126, 1456–1474. doi: 10.1161/CIRCRESAHA.120.317015 32264791 PMC7188049

[B20] Glockner F. O. Yilmaz P. Quast C. Gerken J. Beccati A. Ciuprina A.. (2017). 25 years of serving the community with ribosomal RNA gene reference databases and tools. J. Biotechnol. 261, 169–176. doi: 10.1016/j.jbiotec.2017.06.1198 28648396

[B21] Gupta A. Bhanushali S. Sanap A. Shekatkar M. Kharat A. Raut C.. (2022). Oral dysbiosis and its linkage with SARS-CoV-2 infection. Microbiol. Res. 261, 127055. doi: 10.1016/j.micres.2022.127055 35597076 PMC9065653

[B22] Hirschberger S. Gellert L. Effinger D. Muenchhoff M. Herrmann M. Briegel J. M.. (2022). Ketone bodies improve human CD8(+) cytotoxic T-cell immune response during COVID-19 infection. Front. Med. (Lausanne) 9. doi: 10.3389/fmed.2022.923502 PMC9243504 35783654

[B23] Hoffmann M. Kleine-Weber H. Schroeder S. Kruger N. Herrler T. Erichsen S.. (2020). SARS-coV-2 cell entry depends on ACE2 and TMPRSS2 and is blocked by a clinically proven protease inhibitor. Cell 181, 271–280 e8. doi: 10.1016/j.cell.2020.02.052 32142651 PMC7102627

[B24] Hu C. Rzymski P. (2022). Non-photosynthetic melainabacteria (Cyanobacteria) in human gut: characteristics and association with health. Life (Basel) 12 (4), 476. doi: 10.3390/life12040476 35454968 PMC9029806

[B25] K N. K. Patil P. Bhandary S. K. V. Haridas S. K. N. S. E. Shetty P. (2021). Is butyrate a natural alternative to dexamethasone in the management of CoVID-19? F1000Res 10, 273. doi: 10.12688/f1000research.51786.1 34046165 PMC8108555

[B26] Khanmohammadi S. Rezaei N. (2021). Role of Toll-like receptors in the pathogenesis of COVID-19. J. Med. Virol. 93, 2735–2739. doi: 10.1002/jmv.26826 33506952 PMC8014260

[B27] Kim J. G. Zhang A. Rauseo A. M. Goss C. W. Mudd P. A. O’Halloran J. A.. (2023). The salivary and nasopharyngeal microbiomes are associated with SARS-CoV-2 infection and disease severity. J. Med. Virol. 95, e28445. doi: 10.1002/jmv.28445 36583481 PMC9880756

[B28] Kirst M. E. Li E. C. Alfant B. Chi Y. Y. Walker C. Magnusson I.. (2015). Dysbiosis and alterations in predicted functions of the subgingival microbiome in chronic periodontitis. Appl. Environ. Microbiol. 81, 783–793. doi: 10.1128/AEM.02712-14 25398868 PMC4277562

[B29] Kleinstein S. E. Nelson K. E. Freire M. (2020). Inflammatory networks linking oral microbiome with systemic health and disease. J. Dent. Res. 99, 1131–1139. doi: 10.1177/0022034520926126 32459164 PMC7443998

[B30] Kononen E. Fteita D. Gursoy U. K. Gursoy M. (2022). Prevotella species as oral residents and infectious agents with potential impact on systemic conditions. J. Oral. Microbiol. 14, 2079814. doi: 10.1080/20002297.2022.2079814 36393976 PMC9662046

[B31] Kotrba P. Inui M. Yukawa H. (2001). Bacterial phosphotransferase system (PTS) in carbohydrate uptake and control of carbon metabolism. J. Biosci. Bioeng 92, 502–517. doi: 10.1016/S1389-1723(01)80308-X 16233138

[B32] Li Y. He J. He Z. Zhou Y. Yuan M. Xu X.. (2014). Phylogenetic and functional gene structure shifts of the oral microbiomes in periodontitis patients. ISME J. 8, 1879–1891. doi: 10.1038/ismej.2014.28 24671083 PMC4139721

[B33] Liu Z. M. Yang M. H. Yu K. Lian Z. X. Deng S. L. (2022). Toll-like receptor (TLRs) agonists and antagonists for COVID-19 treatments. Front. Pharmacol. 13. doi: 10.3389/fphar.2022.989664 PMC9518217 36188605

[B34] Louis P. Flint H. J. (2009). Diversity, metabolism and microbial ecology of butyrate-producing bacteria from the human large intestine. FEMS Microbiol. Lett. 294, 1–8. doi: 10.1111/fml.2009.294.issue-1 19222573

[B35] Lu Y. Zhou G. Ewald J. Pang Z. Shiri T. Xia J. (2023). MicrobiomeAnalyst 2.0: comprehensive statistical, functional and integrative analysis of microbiome data. Nucleic Acids Res. 51, W310–W318. doi: 10.1093/nar/gkad407 37166960 PMC10320150

[B36] Ma S. Zhang F. Zhou F. Li H. Ge W. Gan R.. (2021). Metagenomic analysis reveals oropharyngeal microbiota alterations in patients with COVID-19. Signal Transduct Target Ther. 6, 191. doi: 10.1038/s41392-021-00614-3 33986253 PMC8116522

[B37] Miller E. H. Annavajhala M. K. Chong A. M. Park H. Nobel Y. R. Soroush A.. (2021). Oral microbiome alterations and SARS-coV-2 saliva viral load in patients with COVID-19. Microbiol. Spectr. 9, e0005521. doi: 10.1128/Spectrum.00055-21 34643448 PMC8515944

[B38] Miyoshi T. Oge S. Nakata S. Ueno Y. Ukita H. Kousaka R.. (2021). Gemella haemolysans inhibits the growth of the periodontal pathogen Porphyromonas gingivalis. Sci. Rep. 11, 11742. doi: 10.1038/s41598-021-91267-3 34083694 PMC8175725

[B39] Ozilgen M. Yilmaz B. (2021). COVID-19 disease causes an energy supply deficit in a patient. Int. J. Energy Res. 45, 1157–1160. doi: 10.1002/er.5883 33041464 PMC7537131

[B40] Paparo L. Maglio M. A. Cortese M. Bruno C. Capasso M. Punzo E.. (2022). A new butyrate releaser exerts a protective action against SARS-coV-2 infection in human intestine. Molecules 27 (3), 862. doi: 10.3390/molecules27030862 35164139 PMC8838168

[B41] Parks D. H. Tyson G. W. Hugenholtz P. Beiko R. G. (2014). STAMP: statistical analysis of taxonomic and functional profiles. Bioinformatics 30, 3123–3124. doi: 10.1093/bioinformatics/btu494 25061070 PMC4609014

[B42] Ralli T. Saifi Z. Rathee A. Aeri V. Kohli K. (2023). Decoding the bidirectional relationship between gut microbiota and COVID-19. Heliyon 9, e13801. doi: 10.1016/j.heliyon.2023.e13801 36811017 PMC9936796

[B43] Ren Z. Wang H. Cui G. Lu H. Wang L. Luo H.. (2021). Alterations in the human oral and gut microbiomes and lipidomics in COVID-19. Gut 70, 1253–1265. doi: 10.1136/gutjnl-2020-323826 33789966 PMC8042598

[B44] Rognes T. Flouri T. Nichols B. Quince C. Mahe F. (2016). VSEARCH: a versatile open source tool for metagenomics. PeerJ 4, e2584. doi: 10.7717/peerj.2584 27781170 PMC5075697

[B45] Rui L. (2014). Energy metabolism in the liver. Compr. Physiol. 4, 177–197. doi: 10.1002/cphy.c130024 24692138 PMC4050641

[B46] Segata N. Izard J. Waldron L. Gevers D. Miropolsky L. Garrett W. S.. (2011). Metagenomic biomarker discovery and explanation. Genome Biol. 12, R60. doi: 10.1186/gb-2011-12-6-r60 21702898 PMC3218848

[B47] Shobo C. O. Alisoltani A. Abia A. L. K. Mtshali P. S. Ismail A. Zishiri O.. (2020). Bacterial diversity and functional profile of microbial populations on surfaces in public hospital environments in South Africa: A high throughput metagenomic analysis. Sci. Total Environ. 719, 137360. doi: 10.1016/j.scitotenv.2020.137360 32114226

[B48] Teixeira P. C. Dorneles G. P. Santana Filho P. C. da Silva I. M. Schipper L. L. Postiga I. A. L.. (2021). Increased LPS levels coexist with systemic inflammation and result in monocyte activation in severe COVID-19 patients. Int. Immunopharmacol 100, 108125. doi: 10.1016/j.intimp.2021.108125 34543980 PMC8426217

[B49] Thakur K. Tomar S. K. Singh A. K. Mandal S. Arora S. (2017). Riboflavin and health: A review of recent human research. Crit. Rev. Food Sci. Nutr. 57, 3650–3660. doi: 10.1080/10408398.2016.1145104 27029320

[B50] Uehara O. Abiko Y. Nagasawa T. Morikawa T. Hiraki D. Harada F.. (2022). Alterations in the oral microbiome of individuals with a healthy oral environment following COVID-19 vaccination. BMC Oral. Health 22, 50. doi: 10.1186/s12903-022-02093-6 35241064 PMC8892109

[B51] Ventero M. P. Cuadrat R. R. C. Vidal I. Andrade B. G. N. Molina-Pardines C. Haro-Moreno J. M.. (2021). Nasopharyngeal microbial communities of patients infected with SARS-coV-2 that developed COVID-19. Front. Microbiol. 12. doi: 10.3389/fmicb.2021.637430 PMC8010661 33815323

[B52] Wei N. Zhu G. Zhao T. Wang Y. Lou H. Li H.. (2023). Characterization of oral bacterial and fungal microbiome in recovered COVID-19 patients. BMC Microbiol. 23, 123. doi: 10.1186/s12866-023-02872-3 37158877 PMC10166687

[B53] Williams D. W. Greenwell-Wild T. Brenchley L. Dutzan N. Overmiller A. Sawaya A. P.. (2021). Human oral mucosa cell atlas reveals a stromal-neutrophil axis regulating tissue immunity. Cell 184, 4090–4104 e15. doi: 10.1016/j.cell.2021.05.013 34129837 PMC8359928

[B54] World Health Organization COVID-19 epidemiological update - 29 september 2023. Available online at: https://www.who.int/publications/m/item/covid-19-epidemiological-update—29-september-2023 (Accessed 2023-9-29).

[B55] Wu Y. Cheng X. Jiang G. Tang H. Ming S. Tang L.. (2021). Altered oral and gut microbiota and its association with SARS-CoV-2 viral load in COVID-19 patients during hospitalization. NPJ Biofilms Microbiomes 7, 61. doi: 10.1038/s41522-021-00232-5 34294722 PMC8298611

[B56] Xiang Z. Koo H. Chen Q. Zhou X. Liu Y. Simon-Soro A. (2020). Potential implications of SARS-CoV-2 oral infection in the host microbiota. J. Oral. Microbiol. 13, 1853451. doi: 10.1080/20002297.2020.1853451 33312449 PMC7711743

[B57] Xiong D. Muema C. Zhang X. Pan X. Xiong J. Yang H.. (2021). Enriched opportunistic pathogens revealed by metagenomic sequencing hint potential linkages between pharyngeal microbiota and COVID-19. Virol. Sin. 36, 924–933. doi: 10.1007/s12250-021-00391-x 33978940 PMC8114661

[B58] Xu J. Li Y. Gan F. Du Y. Yao Y. (2020b). Salivary glands: potential reservoirs for COVID-19 asymptomatic infection. J. Dent. Res. 99, 989. doi: 10.1177/0022034520918518 32271653

[B59] Xu H. Zhong L. Deng J. Peng J. Dan H. Zeng X.. (2020a). High expression of ACE2 receptor of 2019-nCoV on the epithelial cells of oral mucosa. Int. J. Oral. Sci. 12, 8. doi: 10.1038/s41368-020-0074-x 32094336 PMC7039956

[B60] Yeoh Y. K. Zuo T. Lui G. C. Zhang F. Liu Q. Li A. Y.. (2021). Gut microbiota composition reflects disease severity and dysfunctional immune responses in patients with COVID-19. Gut 70, 698–706. doi: 10.1136/gutjnl-2020-323020 33431578 PMC7804842

[B61] Zhang Z. Yu W. Li G. He Y. Shi Z. Wu J.. (2022). Characteristics of oral microbiome of healthcare workers in different clinical scenarios: a cross-sectional analysis. BMC Oral. Health 22, 481. doi: 10.1186/s12903-022-02501-x 36357898 PMC9648452

[B62] Zhang X. Zhang D. Jia H. Feng Q. Wang D. Liang D.. (2015). The oral and gut microbiomes are perturbed in rheumatoid arthritis and partly normalized after treatment. Nat. Med. 21, 895–905. doi: 10.1038/nm.3914 26214836

[B63] Zhu N. Zhang D. Wang W. Li X. Yang B. Song J.. (2020). A novel coronavirus from patients with pneumonia in China 2019. N Engl. J. Med. 382, 727–733. doi: 10.1056/NEJMoa2001017 31978945 PMC7092803

