# Supplementary material for: Comparative analysis of oropharyngeal microbiota in healthcare workers post-COVID-19
Source: Front Cell Infect Microbiol. 2024 May 17;14:1347345. doi: 10.3389/fcimb.2024.1347345 (PMC11140064; doi:10.3389/fcimb.2024.1347345)
Supplement: Supplementary file 1 [file DataSheet_1.docx]

Supplementary Material

**Supplementary Figures**


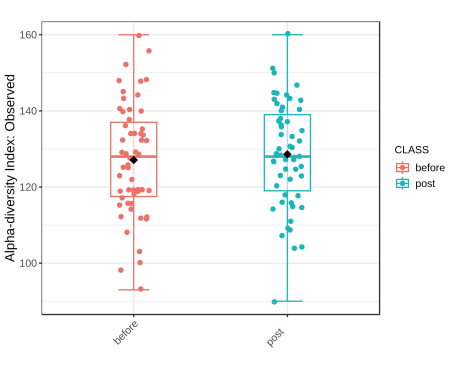

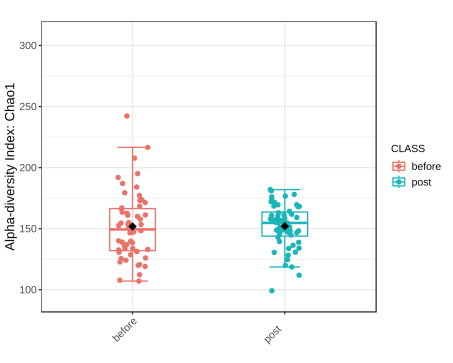
**
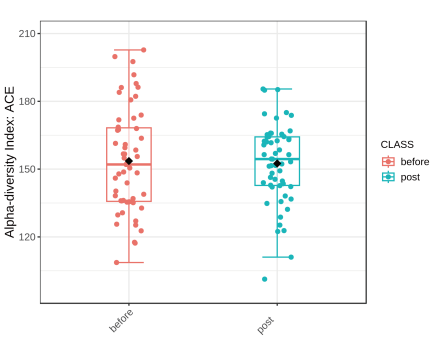
Supplementary Figure 1** Alpha diversity boxplot of the oral microbial community based on the ACE index (P = 0.87), Chao 1 index (P = 0.48), and Richness index (P = 0.55).


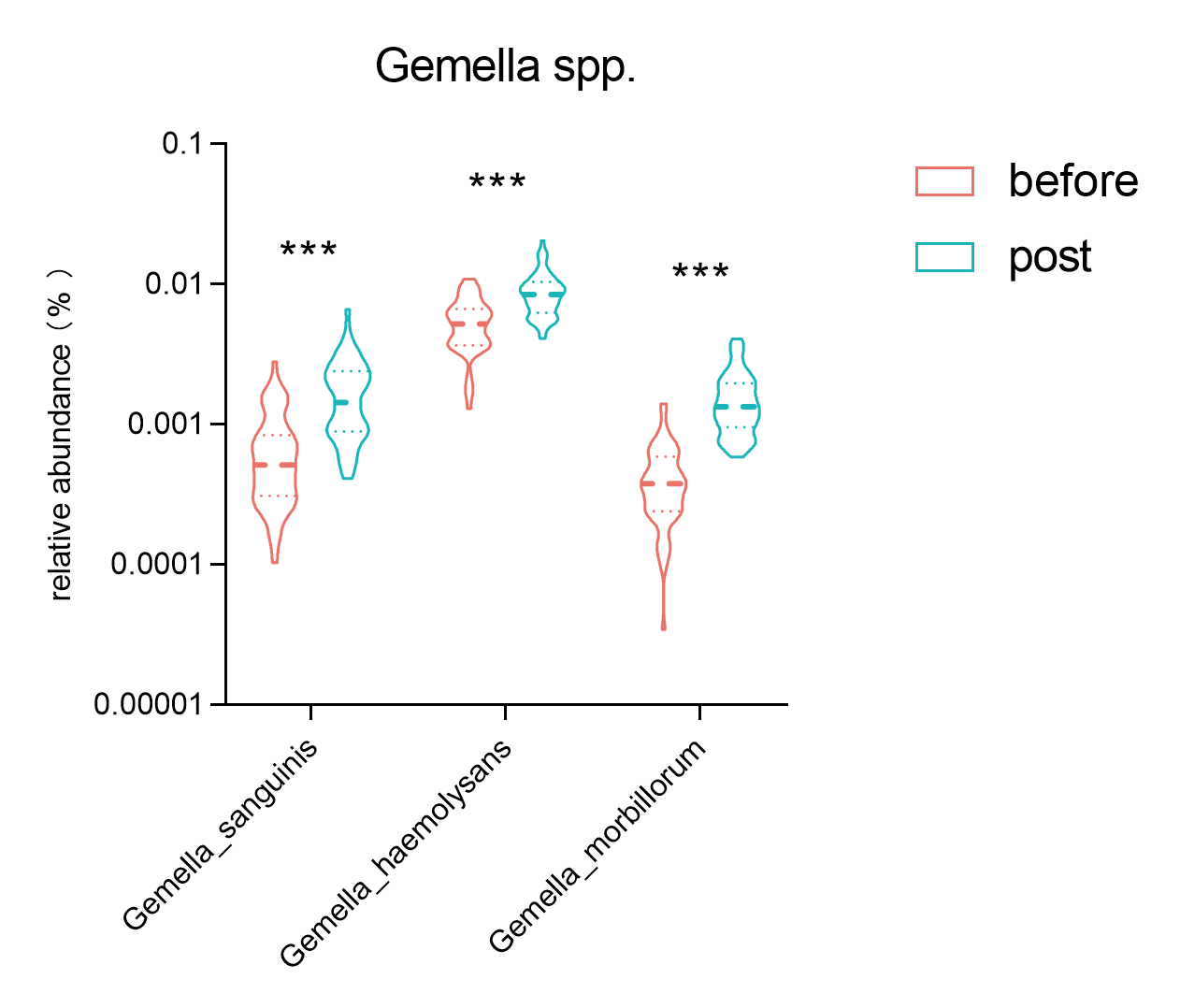


**Supplementary Figure 2** The violin chart reveals the differences in the relative abundance of Gemella spp. in healthcare workers between two groups. *** *P* < 0.001

**Supplementary Tables**

**Supplementary Table 1** Baseline data between mild and moderate disease groups

| Characteristics | Total | Mild | Moderate | *P* value |
| --- | --- | --- | --- | --- |
| Age | 32.76±4.81 | 33.32±4.87 | 32.3±4.8 | 0.4358^a^ |
| Gender |  |  |  | 0.1139^b^ |
| Male | 12 (21.8%) | 8 (32%) | 4 (13.3%) |  |
| Female | 43 (78.2%) | 17 (68%) | 26 (86.7%) |  |
| Seniority | 8.82 ± 4.83 | 8.48±5.09 | 9.1±4.68 | 0.5089^a^ |
| Position |  |  |  | 0.0519^b^ |
| Doctor | 22 (40%) | 14 (56%) | 8 (26.7%) |  |
| Nurse | 33 (60%) | 11 (44%) | 22 (73.3%) |  |

*P* value were calculated using a, kruskal-wallis test; b, chi-square test

**Supplementary Table 2** Baseline data between taste disturbance and normal groups

| Characteristics | Total | Taste disturbance | Normal | *P* value |
| --- | --- | --- | --- | --- |
| Age | 32.76 ± 4.81 | 33.47±5.40 | 32.5±5.40 | 0.5746^a^ |
| Gender |  |  |  | 0.2743^b^ |
| Male | 12 (21.8%) | 5 (33.3%) | 7 (17.5%) |  |
| Female | 43 (78.2%) | 10 (66.7%) | 33 (82.5%) |  |
| Seniority | 8.82 ± 4.83 | 9.93±5.16 | 8.4±4.71 | 0.2941^a^ |
| Position |  |  |  | 0.7582^b^ |
| Doctor | 22 (40%) | 5 (33.3%) | 17 (42.5%) |  |
| Nurse | 33 (60%) | 10 (66.7%) | 23 (57.5%) |  |

*P* value were calculated using a, kruskal-wallis test; b, chi-square test

**Supplementary Table 3** Baseline data between olfactory disorders and normal groups

| Characteristics | Total | olfactory disorders | Normal | *P* value |
| --- | --- | --- | --- | --- |
| Age | 32.76 ± 4.81 | 31.88±4.47 | 33.16±4.97 | 0.4223^a^ |
| Gender |  |  |  | 0.4821^b^ |
| Male | 12 (21.8%) | 5 (29.4%) | 7 (18.4%) |  |
| Female | 43 (78.2%) | 12 (70..6%) | 31 (81.6%) |  |
| Seniority | 8.82 ± 4.83 | 7.76±4.93 | 9.29±4.78 | 0.2432^a^ |
| Position |  |  |  | 0.3764^b^ |
| Doctor | 22 (40%) | 5 (29.4%) | 17 (44.7%) |  |
| Nurse | 33 (60%) | 12 (70.6%) | 21 (55.3%) |  |

*P* value were calculated using a, kruskal-wallis test; b, chi-square test

**Supplementary Table 4** Baseline data for groups with infection duration ≤5d and ＞5d groups

| Characteristics | Total | ≤5d | ＞5d | *P* value |
| --- | --- | --- | --- | --- |
| Age | 32.76 ± 4.81 | 32.74±4.97 | 32.78±4.78 | 0.9966^a^ |
| Gender |  |  |  | 0.5296^b^ |
| Male | 12 (21.8%) | 6 (26.1%) | 6 (18.75%) |  |
| Female | 43 (78.2%) | 17 (73.9%) | 26 (81.25%) |  |
| Seniority | 8.82 ± 4.83 | 8.52±5.09 | 9.03±4.71 | 0.6016^a^ |
| Position |  |  |  | 0.4055^b^ |
| Doctor | 22 (40%) | 11 (47.8%) | 11 (33.3%) |  |
| Nurse | 33 (60%) | 12 (52.2%) | 21 (63.6) |  |

*P* value were calculated using a, kruskal-wallis test; b, chi-square test

**Supplementary Table 5** Baseline data for groups with the interval between the last dose of vaccine before infection and infection ≤1year and ＞1year groups

| Characteristics | Total | ≤1y | ＞1y | *P* value |
| --- | --- | --- | --- | --- |
| Age | 32.76 ± 4.81 | 32.06±4.83 | 33.11±4.83 | 0.9966^a^ |
| Gender |  |  |  | 0.2983^b^ |
| Male | 12 (21.8%) | 2 | 10 |  |
| Female | 43 (78.2%) | 16 | 27 |  |
| Seniority | 8.82 ± 4.83 | 7.22±4.41 | 9.59±4.90 | 0.0802^a^ |
| Position |  |  |  | 0.7709^b^ |
| Doctor | 22 (40%) | 8 | 14 |  |
| Nurse | 33 (60%) | 10 | 23 |  |

*P* value were calculated using a, kruskal-wallis test; b, chi-square test
